# Assessment of Ambient Air Pollution in Istanbul during 2003–2013

**Published:** 2018-08

**Authors:** Eray YURTSEVEN, Suphi VEHİD, Merve BOSAT, Selçuk KÖKSAL, Cemile Nihal YURTSEVEN

**Affiliations:** 1. Public Health Department, Cerrahpasa Faculty of Medicine, Istanbul University, 34320 Kocamustafa Pasa, Istanbul, Turkey; 2. Health Management Department, Faculty of Health Sciences, Istanbul Kemerburgaz University, 34320 Sisli, Istanbul, Turkey; 3. Dept. of Sports Management, Faculty of Sport Sciences, Istanbul University, 34320 Avcılar, Istanbul, Turkey

**Keywords:** Air, Pollution, Particular matter (PM), Health

## Abstract

**Background:**

We investigated the air quality changes from 2003–2013 in Istanbul, Turkey.

**Methods:**

We studied SO_2_, CO and PM concentration patterns in 10 districts of Istanbul. The data was obtained from air pollution monitoring stations of Istanbul Metropolitan Municipality Environmental Protection Department. We compared the variations of mean concentrations monthly, yearly and seasonally. The winter season was accepted from Oct to Mar and the summer season from Apr to Sep.

**Results:**

The highest monthly average values for all measurements of sulfur dioxide and CO were 12.61, 949.19 μg/m^3^ in Sarachane respectively. The Highest value of the monthly average of the Particulate Matter was 72.07 μg/m^3^ in Kartal district. During the period between 2003–2013, monthly mean concentration values of different districts differed significantly in levels of SO_2_ (*P*=0.012), CO (*P*=0.029), and PM (*P*=0.024).

**Conclusion:**

The emissions of air pollutants (SO_2_, PM, and CO) decreased considerably from 2003 to 2013.

## Introduction

That urban air pollution with its long and short-term impacts on human health, well-being, and the environment has been a widely recognized problem during the last 50 years (1–3). In order to protect the atmosphere, governments promote policies and programmes in the areas of energy, efficient transportation, industrial pollution control, and management of toxic and other hazardous wastes. Many countries and different international organizations such as EPA (Environmental Protection Agency), WHO, the European Union Air Quality Framework and Daughter Directives, World Bank, etc. published their own standards for this purpose (4). The economic power of the developed countries, where more than 75% of people live in cities, enables prevention and control of pollution (5). In developing countries, stricter regulations increased the importance of pollution prevention and control particularly in recent years. Moreover, there has been a growing concern about urban air quality in terms of impacts of the pollutants (6,7). However, air pollution is still a serious environmental problem in many urban areas, and many cities in the world suffer from high levels of air pollution. In developing countries, air pollution has been urged upon in recent years because of adverse effects of smog and vehicle exhausts on human. SO_2_, CO and Particular Matter (PM) are the most important indicators for urban air quality assessment (8–14).

Air pollution may harm us. Pollutants accumulate in the air in high concentrations and enter our body in different ways. The cardiovascular and respiratory systems are affected by air pollutants. Effects such as coughing, wheezing, watery eyes arise in relation to air pollution. We inhale around 10000 L of ambient air on a day. Especially sensitive populations (people who are active outdoors, people with lung diseases, children, older adults) are greater risks than the other people. We take more bad breath due to increased air pollution with industrial development. Therefore, pollutants have the potential to affect organs in our body. More than 7 million people die due to the harmful effects of ambient air every year (15). Particulate matter (PM) is produced by a wide variety of natural and anthropogenic activities. Sulfur oxide gasses are formed when fuel containing sulfur is burnt. Exposure to high levels of SO_2_ has been associated with harmful effects such as respiratory illnesses, changes in pulmonary defense and exacerbation of existing cardiovascular disease (16–17). Carbon monoxide is a colorless, odorless, poisonous gas. Carbon monoxide enters the bloodstream and reduces oxygen in the body. The health threat from CO is serious for those who suffer from cardiovascular diseases most. Low working capacities, difficulty in learning, impairment of vision, etc. occur at high CO levels (18–21).

The Istanbul Metropolitan Municipality and The Ministry of Environment and Urbanisation are the main institutions of The Turkish Government that is responsible for the decision-making related to protection of the atmosphere and for transboundary atmospheric pollution control respectively. In order to address the air pollution problem and to plan abatement strategies, the Istanbul Metropolitan Municipality Environmental Protection Department (IMMEPD) has established 10 monitoring networks using the air pollution index (API) to monitor air quality and to provide information on the current air quality.

We studied SO_2_, CO and PM concentration patterns in 10 districts of Istanbul. To facilitate the presentation of the data, we focused on ten years between 2003–2013.

## Methods

### Site description

Istanbul is the most crowded city in Turkey, and it’s also the heart of country’s economy, culture, and history. Starting from the 1970s, the population of Istanbul began to increase rapidly, as people from Anatolia migrated to the city in order to be employed in many new factories constructed on the outskirts of the sprawling metropolis. This sudden sharp rise in the city’s population caused a large demand for housing. Therefore, many previously outlying villages and forests were included by the greater metropolitan area. Istanbul is a transcontinental city located in Eurasia, having its commercial and historical center on the European side and about a third of its population in the Asian side. With a population of 14.4 million, the city forms the largest urban agglomeration in Europe as well as in the Middle East and the sixth-largest city in the world. Istanbul is located in northwestern Turkey on both sides of the Bosphorus strait that connects the Marmara Sea and the Black Sea (11).

### Sampling sites

**Fig. 1:**
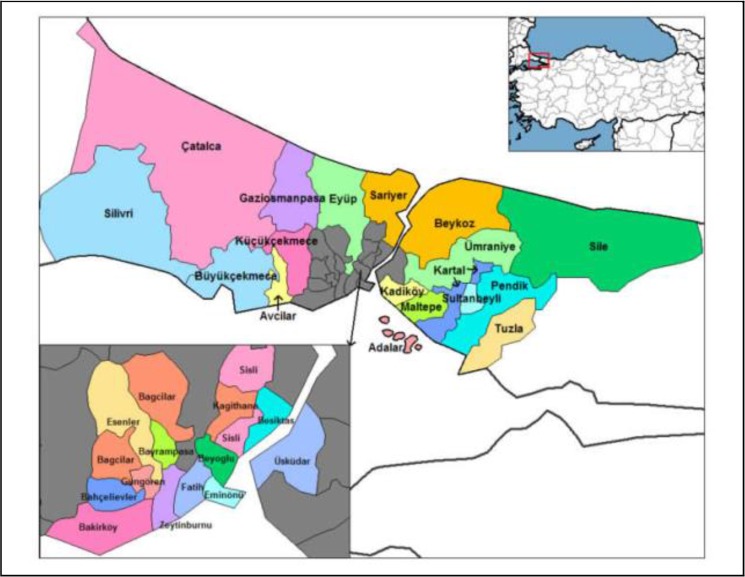
Map showing the districts of Istanbul

In Istanbul, pollutant data were recorded by Istanbul Metropolitan Municipality Environmental Protection Department (IMMEPD) for ten districts. These are Uskudar, Kadikoy, Kartal, Umraniye, Alibeykoy, Besiktas, Esenler, Sarachane, Sarıyer, and Yenibosna. Uskudar, Kadikoy, Kartal, and Umraniye are located in Asia region. Alibeykoy, Besiktas, Esenler, Sarachane, Sarıyer, and Yenibosna are located in Europe region. There were 10 active sampling stations throughout the city and pollutant concentrations were measured. Results were recorded monthly. The locations of selected sampling stations are shown in Fig. 1. This cross-sectional study was carried out between Jan 2003 and Dec 2013 in Istanbul. We aimed to investigate variation in air pollutant parameters (SO_2_, CO, PM) in Istanbul for the dates mentioned above. The data were obtained from air pollution monitoring stations of Istanbul Metropolitan Municipality Environmental Protection Department (IMMEPD). We compared the variations of mean concentrations monthly, yearly and seasonally. While for the winter season, the data from Oct to Mar were detected; for the summer season, the data from Apr to Sep were used. The data were analyzed using SPSS 15.0 (Chicago, IL, USA). Following the statistical evaluation of data and the summarization of descriptive statistics including mean, standard deviation, minimum and maximum values, normality, Student’s t-test and ANOVA Analysis were used.

## Results

The monthly averages of air pollutant parameters (SO_2_, CO and PM) for each year are displayed in Table 1 from 2003 to 2013. The highest monthly average values for all measurements of sulfur dioxide and CO were 12.61, 949.19 μg/m^3^ in Sarachane respectively.

**Table 1:** Average of monthly values of each district in all measurements from 2003–2013

	***Districts***	***N***	***Mean***	***Std. Deviation***	***95% Confidence Interval for Mean***		***Min***	***Max***	***P***
					***Lower Bound***	***Upper Bound***			
SO_2_ (μg/m^3^)	Uskudar	132	7.68	5.34	6.76	8.60	1.00	27.00	0.012
	Kadikoy	132	8.64	6.08	7.59	9.69	1.00	32.00	
	Kartal	132	11.54	8.76	10.03	13.05	1.00	41.00	
	Umraniye	130	8.40	7.29	7.14	9.67	1.00	38.00	
	Alibeykoy	132	11.09	8.58	9.61	12.57	1.00	42.00	
	Besiktas	132	12.47	8.98	10.92	14.02	1.00	38.00	
	Esenler	132	11.09	8.96	9.55	12.63	1.00	52.00	
	Sarachane	132	12.61	8.08	11.22	14.00	1.00	47.00	
	Sarıyer	132	8.72	7.53	7.42	10.02	1.00	39.00	
	Yenibosna	132	12.16	9.81	10.47	13.85	1.00	41.00	
PM (μg/m^3^)	Uskudar	132	37.59	11.55	35.60	39.58	13.00	82.00	0.024
	Kadikoy	132	48.16	14.46	45.67	50.65	14.00	96.00	
	Kartal	132	72.07	19.12	68.78	75.36	26.00	142.00	
	Umraniye	132	46.29	12.32	44.17	48.41	16.00	93.00	
	Alibeykoy	131	57.08	20.07	53.62	60.54	12.00	130.00	
	Besiktas	132	51.55	27.71	46.78	56.32	21.00	331.00	
	Esenler	131	62.15	18.38	58.97	65.33	26.00	132.00	
	Sarachane	132	59.36	15.22	56.74	61.98	23.00	96.00	
	Sarıyer	132	43.63	13.89	41.24	46.02	20.00	90.00	
	Yenibosna	132	58.17	14.06	55.75	60.59	24.00	110.00	
CO (μg/m^3^)	Uskudar	132	671.44	282.93	622.74	720.24	170.00	2292.00	0.029
	Kadikoy	132	741.64	342.78	682.64	800.74	203.00	1904.00	
	Kartal	132	779.40	295.08	728.64	830.24	246.00	2152.00	
	Umraniye	132	638.93	258.72	594.43	683.53	7.00	1516.00	
	Alibeykoy	132	663.83	349.47	603.73	724.03	27.00	2424.00	
	Besiktas	132	853.59	261.44	808.69	898.69	452.00	1748.00	
	Esenler	132	686.51	264.75	640.91	732.11	293.00	1839.00	
	Sarachane	131	949.19	469.91	868.19	1030.19	55.00	3963.00	
	Sarıyer	132	585.82	241.10	544.32	627.32	174.00	1459.00	
	Yenibosna	132	613.73	217.19	576.33	651.13	161.00	1301.00	

The highest value of the monthly average of the Particulate Matter was 72.07 μg/m^3^ in Kartal district. During the period between 2003–2013, monthly mean concentration values of different districts differed significantly in levels of SO_2_, CO and PM (Table 1).

The annual averages for all years in air pollutant parameters (SO_2_, CO and PM) are displayed in Table 2 in the period between 2003–2013. The highest value in all measurements of sulfur dioxide, PM and CO were measured at 17.88±10.05, 58.51±17.36, 935.84±510 μg/m^3^ in 2003 respectively. During the period between 2003–2013, values of the annual mean concentrations differed significantly in levels of SO_2_, CO and PM (Table 2).

**Table 2:** Annual averages value in all measurements from 2003–2013

		***N***	***Mean***	***Std. Deviation***	***95% Confidence Interval for Mean***		***Min***	***Max***	***P***
					***Lower Bound***	***Upper Bound***			
SO_2_ (μg/m^3^)	2013	120	3.54	1.68	3.24	3.85	1	9	0.036
	2012	120	4.93	2.45	4.48	5.37	1	13	
	2011	120	6.25	3.48	5.62	6.88	1	16	
	2010	118	6.46	3.41	5.84	7.08	1	19	
	2009	119	9.23	5.60	8.21	10.24	1	30	
	2008	120	7.48	5.17	6.54	8.41	1	22	
	2007	120	13.11	7.73	11.71	14.51	1	35	
	2006	120	15.34	8.03	13.89	16.79	2	36	
	2005	120	13.68	9.63	11.94	15.42	1	41	
	2004	120	16.94	8.76	15.36	18.52	1	40	
	2003	120	17.88	10.06	16.07	19.70	1	52	
PM (μg/m^3^)	2013	120	55.73	16.63	52.72	58.73	20	102	0.029
	2012	120	52.48	15.34	49.71	55.26	24	109	
	2011	120	48.84	15.28	46.08	51.60	22	109	
	2010	120	49.87	15.27	47.11	52.63	22	96	
	2009	119	54.83	33.74	48.71	60.96	16	331	
	2008	120	56.91	16.65	53.90	59.92	22	100	
	2007	120	57.22	15.93	54.34	60.10	23	113	
	2006	119	57.64	25.54	53.00	62.28	18	142	
	2005	120	48.38	18.00	45.12	51.63	12	111	
	2004	120	49.26	15.65	46.43	52.09	19	96	
	2003	120	58.51	17.37	55.37	61.65	21	107	
CO (μg/m^3^)	2013	120	634.66	182.08	601.75	667.57	169	966	0.022
	2012	120	671.75	137.86	646.83	696.67	293	1.054	
	2011	120	643.32	195.08	608.05	678.58	56	1.192	
	2010	119	630.91	207.26	593.28	668.53	30	1.103	
	2009	120	645.13	269.67	59639	693.88	174	1.621	
	2008	120	603.27	240.32	559.83	646.71	161	1.258	
	2007	120	727.60	253.26	681.82	773.38	7	1.432	
	2006	120	816.39	411.04	742.09	890.69	199	2.424	
	2005	120	757.07	360.69	691.87	822.26	27	1.737	
	2004	120	833.96	398.71	761.89	906.03	219	2.152	
	2003	120	935.84	510.82	843.51	1028.18	280	3.963	

Table 3 shows seasonal average values of the air pollutants in all measurements from 2003–2013. Values, recorded in winter, were higher than those recorded in summer for all pollutants. There were statistically significant differences between the seasons.

**Table 3:** Seasonal average values in all measurements from 2003–2013

	***Seasons***	***N***	***Mean***	***Std. Deviation***	***Std. Error Mean***	***T***	***P-value***
SO_2_ (μg/m^3^)	Winter	658	13.03	9.14	0.35	12.005	0.038
	Summer	659	7.86	6.19	0.24		
PM (μg/m^3^)	Winter	660	57.43	21.33	0.83	7.189	0.025
	Summer	658	49.75	17.17	0.66		
CO (μg/m^3^)	Winter	659	861.69	334.64	13.03	17.93	0.014
	Summer	660	57500	237.82	9.25		

Table 4 shows that there were statistically significant differences between the European and Asian sides of the city for the pollutants SO_2_ and PM. There was no significant difference for carbon monoxide (*P*>0.05).

**Table 4:** Continental average values in all measurements from 2003–2013

	***Continent***	***N***	***Mean***	***Std. Deviation***	***Std. Error Mean***	***T***	***P-value***
SO_2_	Europe	791	11.36	8.76	0.31	4.990	0.022
	Asia	526	9.07	7.12	0.31		
PM	Europe	790	55.31	19.76	0.70	3.880	0.015
	Asia	528	51.03	19.43	0.84		
CO	Europe	791	725.16	338.05	12.02	0.952	0.870
	Asia	528	707.85	300.79	13.09		

**Fig. 2:**
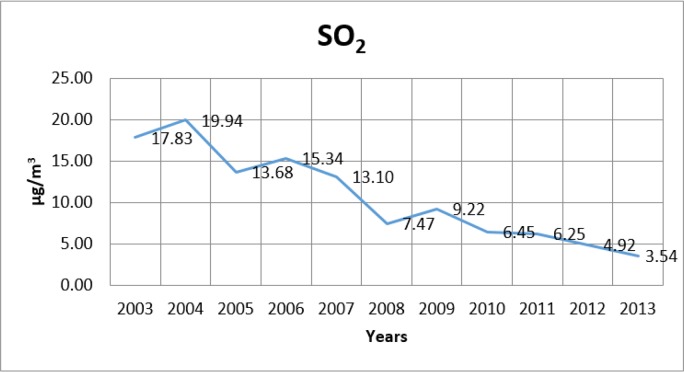
Change of SO_2_ levels in Istanbul by years

**Fig. 3:**
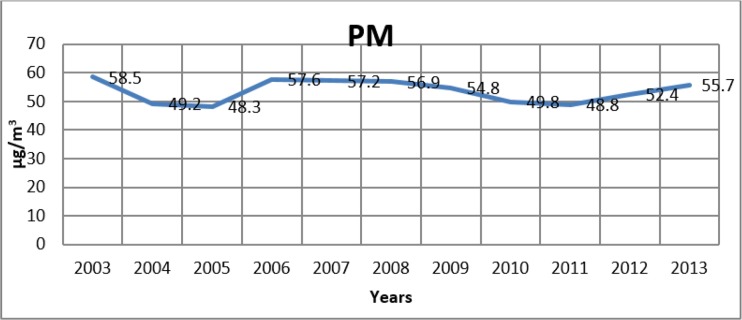
Change of PM levels in Istanbul by years

**Fig. 4:**
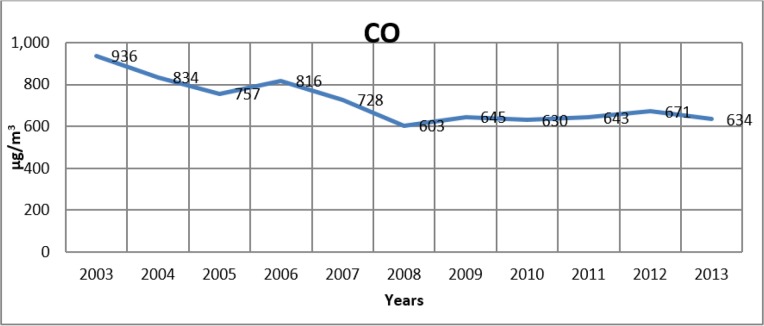
Change of CO levels in Istanbul by years

Figures 2–4 show the change in the annual average values of SO_2_, PM and CO (μg/m^3^) in Istanbul for the years from 2003–2013.

## Discussion

During the period from 2003–2013 for monthly mean values, Uskudar was the lowest concentration for the SO_2_ and PM 7.68±5.34, 37.59±11.55 respectively. The highest monthly mean value for SO_2_ was found to be 12.61±8.08 μg/m^3^ in Sarachane district. The highest values were measured for particulate matter 72.07±19.12 μg/m^3^ in Kartal district. The study performed by Elber et al. showed 68 μg/m^3^ in Istanbul (22). This result is the same as ours. The reason why Uskudar is at the low levels of pollutants is that of its being located near the coast of Asian side and also because there is no big industrial area near it. SO_2_ and PM were significantly associated with total non-accidental mortality and respiratory mortality in cities. Our results demonstrate that SO_2_ and PM levels were below the Turkish and European air quality guidelines (23). SO_2_ was the most significantly associated pollutant with cardiovascular, respiratory and natural mortality in the city. If we compare the results of our study with different European studies, these estimates are consistent with the direction of the association. Zmirou, Stieb López-Villarrubia and reported the existence of consistent associations between SO_2_ levels and cardiovascular and respiratory mortality (24–26).

With the rapid economic and industrial development increase in emission sources in İstanbul. The number of vehicles is increasing continuously in Istanbul. Therefore, promoting alternative and more sustainable modes of transport to the cars is one of the most important requirements for air pollution reduction in the city. The change in the air quality has particular importance for several reasons. For example, policy-makers are highly interested in the effectiveness of regulations and are enacted to reduce the emissions of gaseous and solid species. They expect a lower level of ambient air pollution. It is not due to emissions only; as it is in accordance with the causal chain of air pollution as well (27). The assessment summarised the measured data into the least possible number of factors that characterized the overall ambient air quality within 2003–2013. We have found that pollutant values were recorded in winter seasons were higher than that of summer seasons. Summer and winter periods for pollutants evaluated include statistically significant difference was found. All pollutant (SO_2_, CO and PM) levels were higher during winters because of the widespread usage of fossil fuels. As winter seasons were more dangerous for air pollution, we recommend legal authorities to monitor winter more carefully. A similar conclusion was reported dating back to 2008 (28). When the pollutant levels compared intercontinental (Asia and Europe), SO_2_ and PM levels were significantly different but CO was not (*P*>0.05). We thought that this difference was due to the fact that European region has more population and excessive traffic.

The levels of SO_2_ (one of the main pollutants released by burning fossil fuels) and CO (resulting from incomplete combustion) decreased from 2003 to 2013. “One reason for this decline may be the widespread adoption of natural gas in Istanbul, starting from the millennium” (11). When it comes to other reasons, while one of them is the increase in the quality standards of the coal and other fossil fuels used in the city, the other one is the enforcement of regulations limiting the use of coal containing high levels of sulfur. “The mean SO_2_ concentration between 2002 and 2010 declined by 21% on the European side of Istanbul and 28% on the Asian side” (11). In our study, the annual mean SO_2_ concentration from 2003–2013 declined by %80 in all districts. The annual changes in CO concentration are similar to those of SO_2_. The annual mean concentrations of CO decreased from the year 2003 to 2013. These findings were similar to other studies (11).

## Conclusion

Our study investigated the changes in SO_2_, PM and CO emissions in ten districts of Istanbul over the period from 2003 to 2013. The emissions of air pollutants (SO_2_, PM, and CO) decreased considerably from 2003 to 2013. Legal authorities should continue to measure and control air pollutant levels frequently for this improvement to continue. Air pollutants are higher especially in the winter season and that is why the content of the fuel should be more tightly regulated by officials.

## Ethical considerations

Ethical issues (Including plagiarism, informed consent, misconduct, data fabrication and/or falsification, double publication and/or submission, redundancy, etc.) have been completely observed by the author.
